# Mechanical Properties of Natural Rubber Filled with Foundry Waste Derived Fillers

**DOI:** 10.3390/ma12111863

**Published:** 2019-06-09

**Authors:** Ting Xie, Fajun Wang, Chan Xie, Sheng Lei, Shijin Yu, Jiawei Liu, Daqi Huang

**Affiliations:** 1 School of Materials Science and Engineering, Nanchang Hangkong University, Nanchang 330063, China; mingshanxue@163.com (T.X.); jjbxsjz@163.com (C.X.); 2 School of Materials Engineering, Jiangsu University of Technology, Changzhou 213001, China; shenglei@just.edu.cn; 3 Shiyan Litong New Materials Technology Co., LTD, Shiyan 442599, China; bigwfj@foxmail.com; 4 School of Mechanical and Electronic Engineering, Jingdezhen Ceramic Institute, Jingdezhen 333403, China; yushijin@163.com (S.Y.); yingzhi110@163.com (J.L.)

**Keywords:** foundry dust, natural rubber, waste disposal, tensile strength, tear strength

## Abstract

The main aim of this study is to evaluate the possibility of applying foundry dust (FD) derived filler for the preparation of natural rubber (NR) based composites by characterizing the mechanical properties. The as-received FD was processed via a simple and low-cost procedure, including sieving, deironing and milling using a variety of industrial equipment. FD powders before and after silane coupling agent (Si 69) modification were used as fillers for NR. NR composites inserted with different content of modified and unmodified FD up to 50 phr were prepared via dry-mixing method. Then, comprehensive mechanical performances were performed on the corresponding vulcanizates. It was demonstrated that NR composite filled with 50 phr of modified FD exhibited optimized comprehensive mechanical performance. Tear strength and hardness is increased by 21.3% and 12.8% than pure NR, respectively. Tensile strength is reduced by 21% and elongation at break remained nearly unchanged. Additionally, the composite showed a large increment of 50.9% for its wet grip property, while exhibited an increment of only 11.9% for its rolling resistance in comparison with the composite containing 10 phr of FD. The findings of this study may provide a new application area for the large amounts of utilization of foundry waste with a high level of value being added.

## 1. Introduction

A large amount of foundry waste has been produced in the foundry industry, including waste foundry sand, slag, dust, etc. [1,2,3]. China is the largest casting producing country in the world. There are more than 20,000 foundries in China, generating 20–30 million tons of foundry waste annually [4]. It was reported that foundry wastes have some application areas, such as fine and/or coarse fillers in concrete, cement production, bricks, embankment material and road bases [5,6,7,8]. However, no more than 15% of foundry waste has been reused in these areas [9]. The common method for disposing of foundry waste is to pile it up in landfills, which creates a significant waste of resources and potential environmental pollution [8]. In addition, the conventional method of landfilling has become increasingly difficult because of increasing transportation cost, scarcity of land, as well as stringent environmental laws and regulations [2]. Therefore, it is very urgent to develop new methods for the utilization of foundry waste, especially in those application areas that foundry waste can be used in large quantities. We expect that rubber products containing foundry waste derived fillers could resolve this problem.

The total global demand for rubber materials in 2017 is 27.1 million tons, including 12.5 million tons of NR and 14.63 million tons of synthetic rubber [10,11,12]. Rubber materials were mainly composed of rubber matrix, reinforcing fillers and other additives. The total content of reinforcing filler in rubber composites is usually greater than 50% [13,14,15]. Carbon black and silica are the most used reinforcing fillers for rubber composites. However, carbon black is a non-renewable material that is derived from petroleum, and the preparation of silica involves harsh chemicals. The utilization of renewable materials and/or waste-derived materials possessing good reinforcing and processing properties, as well as being of low cost, is highly desired in the rubber industry [13,14,15,16]. Barrera et al. reinforced natural rubber with eggshell, tomato peels, carbon fly ash and guayule bagasse, respectively. Some of the obtained composites showed significant increase of both tensile strength and tear strength compared to pure nature rubber. In addition, the power consumption during compounding decreased by up to 19% in the composites [10]. Yu et al. used lignin/silica hybrid filler to reinforce the nature rubber, the vulcanizate possessed optimal properties containing 30 phr of silica and 20 phr lignin [16]. Siriwong et al. reinforced natural rubber with a coupling agent treated with spent coffee grounds. It was observed that the obtained composite exhibited higher hardness and modulus compared to that of the untreated one [17]. However, to the best of our knowledge, rubber-based composites applying foundry waste-derived filler as a reinforcing agent have not been reported. 

**Figure 1:**
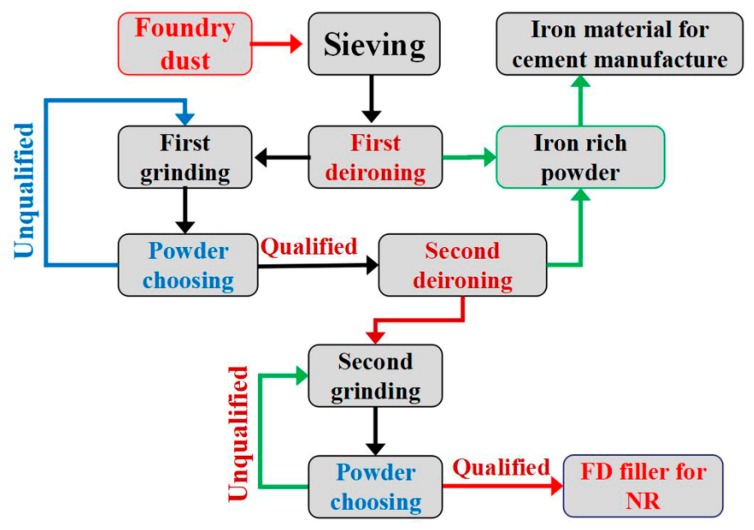
Schematic diagram of processing foundry dust derived fillers.

The main aim of this study is to evaluate the possibility of applying waste derived filler for the production of NR composites. Among various foundry wastes, we chose foundry dust as raw material because its particle size is significantly lower than that of foundry waste sand and slag [18]. Before use, the foundry dust powder was processed through a simple separation and grinding process as depicted in Figure 1. The processing cost is only 400 Chinese yuan per ton. The particle size and distribution, chemical component and surface modification were characterized using laser particle size analysis, X-ray fluorescence analysis and infrared spectrum analysis, respectively. Subsequently, the mechanical properties were evaluated using static tensile tests and dynamic mechanical performance tests. The use of foundry waste as filler for rubber products is of great importance because it could not only provide a new application area for foundry waste, but also lower the cost of rubber product.

## 2. Materials

Foundry dust was kindly provided by Yunxian Litong Renewable Resources Processing Co. LTD (Shiyan, China). Natural Rubber (NR, type WF) was purchased from Hengshui New Antai Chemical co. LTD. Bis-(3-[triethoxysilyl]-propyl)-tetrasulfide (Silane coupling agent, Si 69) was purchased from Aladdin Company (Shanghai, China). Fumed Silica (FS, type M5) was obtained from Shanghai Cabot chemical co. LTD. Other rubber additives, such as stearic acid (SA), zinc oxide (ZnO), Sulfur (S), N-isopropyl-N-phenyl-phenylenediamine (Antioxidant 4010NA), 2,2′-Dibenzothiazole disulfide (accelerant DM), were obtained from Aldrich Company (Shanghai, China). Except for the foundry dust, all of the above materials and reagents were used as received without any treatment.

## 3. Preparation

The original foundry dust was collected from the dust collector of the local foundry. The foundry dust was processed in Yunxian Litong Renewable Resources Processing Co. LTD (Shiyan, China) before use. As shown in Figure 1, the coarse particles were separated using an industrial vibrating screen (80 mesh). Subsequently, the magnetic substance (e.g., Fe_2_O_3_) was preliminarily separated using a roller type magnetic separator. Then an ultra-fine powder mill with the function of powder selection was used to grind the foundry dust in order to obtain fine particles. The qualified powder was subjected to the secondary removal of iron, while the unqualified powder was sent into the mill and ground again. It should be mentioned that the obtained magnetic substances (iron-rich material) were collected and could be used for the manufacturing of cement. After deironing twice, the foundry dust powders were milled again until the suitable particle size was obtained. The obtained foundry dust powders are abbreviated as FD hereafter.

The FD powder was modified with Si 69. Briefly, 100 g FD powder was dispersed in 500 mL ethanol under vigorous mechanical stirring. Then, 0.5 g Si 69 and 1 mL water were added into the dispersion, respectively. The dispersion was continuously stirred for 2 h. After filtration and drying, silane modified FD powder was obtained.

The obtained dust powder was modified with Si 69 before use. Briefly, 50 g of FD powder was dispersed in n-hexane (500 mL) in a beaker under drastic agitation. Subsequently, 1.5 g of Si 69 was added into the above dispersion. The dispersion was stirred continuously for 2 h. Finally, the modified FD powder was obtained after filtration, followed by drying. The modified powders of FD are abbreviated as mFD.

The NR composites with different filler content were compounded on the two-roll open mill (SK-160, Yangzhou Yuanfeng Test Machinery Factory, Yangzhou, China) around ambient temperature. The NR was first laminated for 3 min on the mill. Then ZnO, SA, 4010NA, reinforcing agent (FD or mFD), DM and S were added into the NR successively. The compositions of the NR composites are shown in Table 1. After being homogeneously blended, the mixtures were thin-passed for 5 t and stored at ambient environment for about 48 h. The mixtures were hot-molded at 160 °C for an optimal curing time (t_90_) using Plate vulcanizer (YF-8017, Yangzhou Yuanfeng Test Machinery Factory, China). For convenience, the NR composites containing different filler content (phr) were abbreviated as 10 FD/NR, 20 FD/NR, 30 FD/NR, 40 FD/NR, 50 FD/NR, 10 mFD/NR, 20 mFD/NR, 30 mFD/NR, 40 mFD/NR and 10 mFD/NR, respectively.

## 4. Characterization and Measurement

The time of optimal curing (t_90_) was measured using a non-rotor rheometer (YF-8105, Yangzhou Yuanfeng Test Machinery Factory, Yangzhou, China). Tensile properties (standard ISO 37-2005) and tear strength (standard ISO 34-1:2004) were performed using a tensile machine (YF-900, Yangzhou Yuanfeng Test Machinery Factory, Yangzhou, China) at room temperature. Shore A hardness (according to the standard ISO 7619-1:2004) was measured using a durometer (LX-A, Yangzhou Yuanfeng Test Machinery Factory, Yangzhou, China). Energy storage modulus and loss factor as a function of temperature were measured using a dynamic mechanical thermal analyzer (DMA, Netzsch DMA 242).

The particle size distribution of the FD powders was analyzed using a laser particle size analyzer (Mastersizer 3000, Malvern Panalytical, UK). The element content of the FD powders were analyzed using an X-ray fluorescence spectrometer (EDX-7000, Shimadzu, Japan). The fractured surface of the NR composites was measured using a Field Emission Scanning Electron Microscope (FE-SEM, FEI NanoSEM-430, FEI Corporation, Hillsboro, OR, America). The particles of FD and mFD were analyzed using a Burker Vertex 33 infrared spectrometer in the range of 400–4000 cm^−1^.

## 5. Results and Discussion

**Figure 2:**
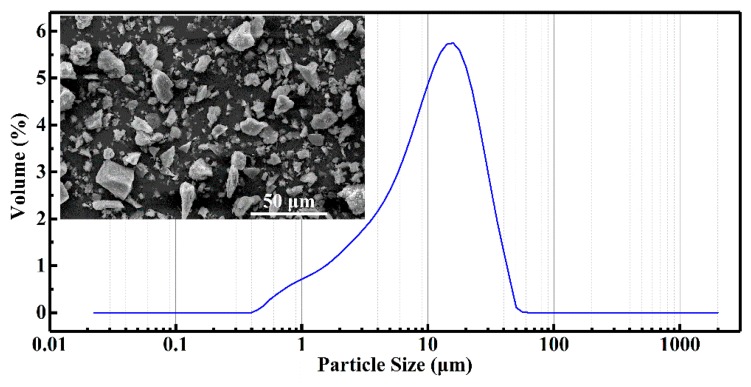
The particle size distribution of the obtained foundry dust (FD) powders. The inset shows the FE-SEM image of the FD powder.

Figure 2 shows the particle size distribution of the obtained FD powders. One can see that most of the particles (above 99.95 vol.%) are between 0.4 μm and 50 μm. The fine particles with size lower than 10 μm have a volume percentage of about 53.25% and the coarse particles (larger than 20 μm) are only 14.05 vol.%. In addition, the middle-sized particles with diameter ranging from 10 μm to 20 μm are about 37.86% in volume. The results are in accordance with the FE-SEM measurement, which is shown in the inset of Figure 2. It is clearly observed that the particles with irregular shapes and various sizes are uniformly distributed in the FE-SEM image. The X-ray Fluorescence spectroscopy (XRF) measurement of the FD powders are summarized in Table 2. It is observed that the main elements in the FD powders are O (49.00 wt.%), Si (33.74 wt.%), Al (6.59 wt.%), Ca (2.64 wt.%) and Fe (2.40 wt.%). The corresponding weigh percentage of the above elemental oxides are 72.17% for SiO_2_, 12.51% for Al_2_O_3_, 3.66% for CaO, and 3.43 for Fe_2_O_3_, respectively. The total content of the above four oxides are as high as 91.77 wt.%. These oxides are mainly in the form of quartz, alumina, silicate and aluminosilicate during the casting process [18,19,20].

**Figure 3:**
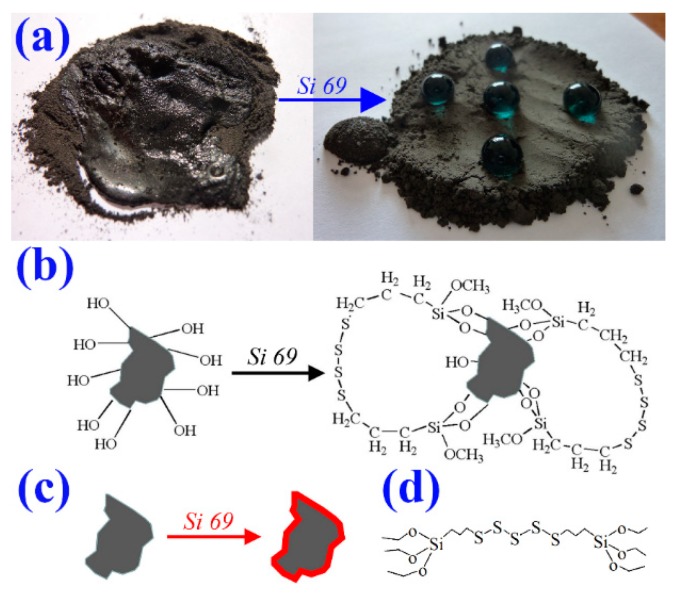
(**a**) Digital photos of the wettability measurement for FD powders before and after Si 69 modification. Left: water spreads over the surface of FD powders; right: water droplets stand on the surface of modified powders of foundry dust (mFD) powders. Water was dyed blue for clear observation; (**b**) chemical reaction between FD particles and Si 69; (**c**) the simplified expression of (**b**); (**d**) the chemical structure of Si 69.

**Figure 4:**
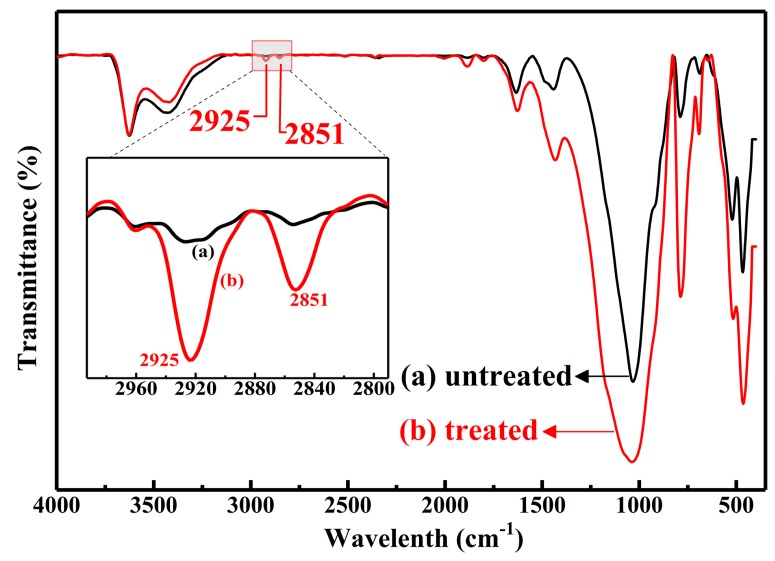
FT-IR specta of FD and mFD particles.

Generally speaking, most of the oxides are intrinsically hydrophilic because of the surface polar groups of hydroxy, while NR is a non-polar polymer [19,20,21]. As a result, the polar powders of inorganic oxides cannot be well dispersed in the NR matrix. In addition, the combination between fillers and polymer chains are relatively weak due to lacking a chemical bond. Both of these factors lead to the deterioration of mechanical properties. The wettability of the powder surface of FD and mFD was examined by adding water droplets on the powder surface (see Figure 3a). Water spread over the powder surface of FD, indicating the hydrophilicity of the surface. However, the surface exhibits hydrophobicity after being modified with Si 69. One can see that water droplets show spherical shapes and stand on the powder surface (see Figure 3a). The chemical reaction between FD particles and Si 69 was briefly described in Figure 3b. The relevant reaction process is described as follows: Firstly, the methoxyl group of Si 69 could react with water (adsorbed from air) and generate hydroxyl groups Figure 3d. Secondly, the hydroxyl groups of Si 69 react with the hydroxyl groups on the surface of FD particles. After a period of time, an organic layer is formed on the particle surface of FD Figure 3c. The organic layer is non-polar and possesses low surface free energy, which is responsible for the surface hydrophobicity of the mFD powders, as shown in Figure 3a [21,22,23,24]. The surface bonded organic layer could be further verified by FT-IR measurement. Figure 4 shows the FT-IR spectra of FD particles before and after the modification of Si 69. It is clearly observed that two new absorption peaks appeared after the modification of silane. The absorption peak at wave number of 2925 cm^−1^ and 2851 cm^−1^ are responsible for the asymmetrical stretching vibration and symmetrical stretching vibration of CH_2_ group respectively, which is derived from Si 69 [25,26]. From the surface wettability observation, combined with the FT-IR measurement, we can conclude that the molecule of Si 69 is successfully modified on the surface of FD particles.

**Figure 5:**
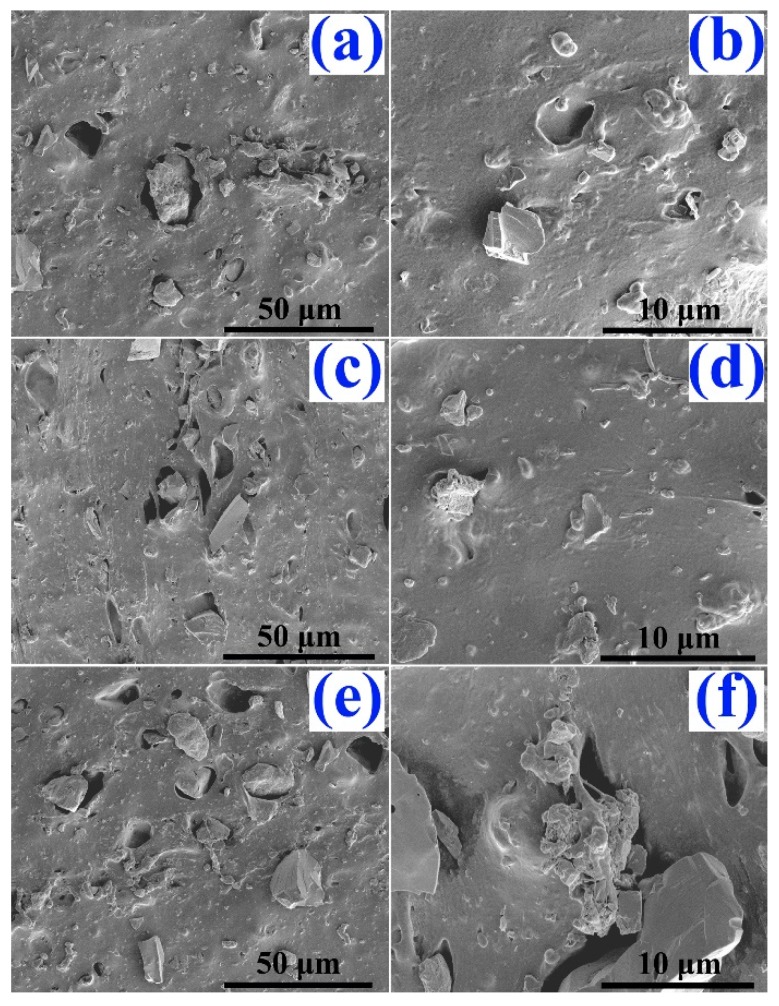
FE-SEM images of the fractured surface of the NR composites with different FD content (phr). (**a**) and (**b**) phr is 10; (**c)** and (**d**) phr is 30; (**e**) and (**f**) phr is 50.

**Figure 6:**
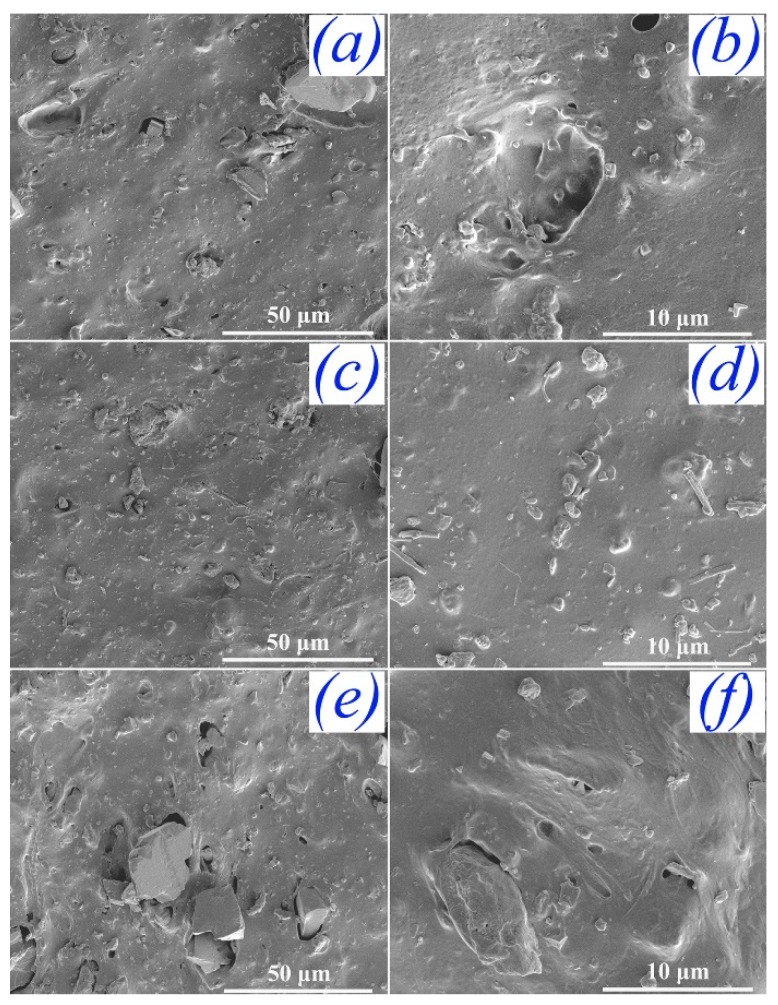
FE-SEM images of the fractured surface of the NR composites with different mFD content (phr). (**a**) and (**b**) phr is 10; (**c**) and (**d**) phr is 30; (**e**) and (**f**) phr is 50.

After the characteristics of FD-derived filler powders, such as the particle size and the distribution, chemical composition, surface wettability and surface chemistry were carefully studied, we attempt to investigate the performance of NR composites filled with FD. It was reported that the distribution of fillers in the rubber matrix and the interfacial interactions between rubber matrix and filler played important roles in determining the mechanical performance. The fractured micro-structures of the FD/NR composites were observed and are depicted in Figure 5. It can be clearly observed that the continuous phase is the NR matrix and the irregular particles are FD. The obvious agglomerate of fillers can also be seen in Figure 5a,d,f. The FD particles without silane modification are highly polar, while the natural rubber is a non-polar material. There is a great difference in surface energy between the two materials. The difference of surface energy will lead to poor compatibility and uneven distribution of powders and rubber, resulting in agglomeration and voids of powders. Additionally, there are many pores existing on the fractured surface of the composites (see Figure 5a–c,f). Comparative FE-SEM studies are shown in Figure 6. The micro-structures of the mFD/NR composites are clearly improved. One can see that the mFD particles are uniformly distributed in the NR matrix and no obvious pores can be observed (see Figure 6a–d) when the content of mFD is relatively low (10 and 30 phr). Though pores appear in the composite of 50 mFD/NR, the pores are very small and the total amount of pores in the composite is much less than that in FD/NR composite.

The vulcanization parameters of the mFD/NR composites are depicted in Table 3. One can see that with the increase of mFD content, the torque of raw rubber increases and the curing time prolongs. CB/NR composite with CB (N330) content of 50 phr were used for a reference. The MH-ML and tc90 are 1.58 dN·m and 268 s respectively. Therefore, the torque of the mFD/NR composite is much smaller than that of the CB/NR composite, indicating that the mFD has better processing properties than CB. In addition, the curing time difference between the two kinds of composite materials is not significant, which indicates that the two fillers do not adversely affect the vulcanization of the rubber when used together. The combination of various fillers is beneficial to the improvement of comprehensive properties of rubber composites.

**Figure 7:**
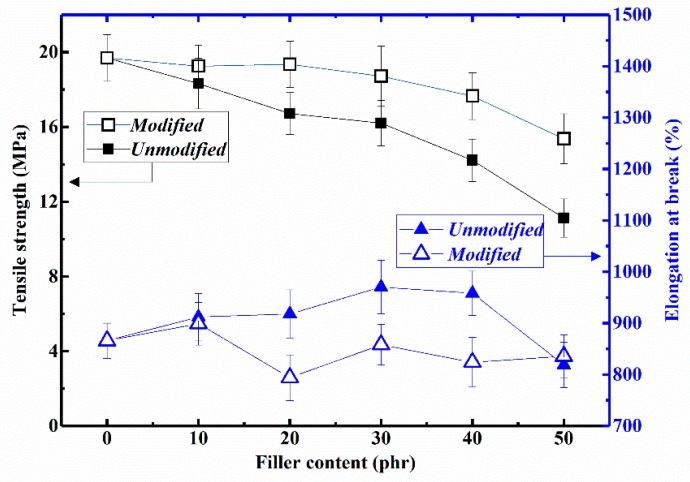
The influence of filler content on the tensile strength and elongation at break of the FD/NR and mFD/NR composites.

Figure 7 shows the tensile strength and elongation at break of the FD/NR and mFD/NR composites. The tensile strength of unreinforced natural rubber after vulcanization is about 19.69 MPa. After incorporated with FD or mFD, the tensile strength of both FD/NR and mFD/NR composites decrease gradually with increasing filler content. It should be mentioned that the tensile strength of the mFD/NR composite is always larger than that of the FD/NR composite filled with the same filler content. The enhanced tensile strength of the FD/NR composite should be attributed to the surface modification of Si 69 of FD, which is beneficial to reduce the aggregation of FD in NR phase and increase the interfacial combination between filler and NR matrix (see Figure 3, Figure 5 and Figure 6). It is very desirable for practical application that the mFD/NR composite with high filler content (50 phr) still exhibits a relatively high tensile strength (15.36 MPa). Filling high concentrations of foundry dust in rubber not only increases the output of rubber products, but also reduces the cost. More importantly, this process utilizes casting waste, which has important social and environmental benefits. In addition, the elongation at break of the composites varies in the range of 800% to 1,000%, which shows that the addition of FD has no negative impact on the elongation property of the composites. Typically, the FD/NR composite with 50 phr of mFD exhibits a high elongation at break around 835%.

**Figure 8:**
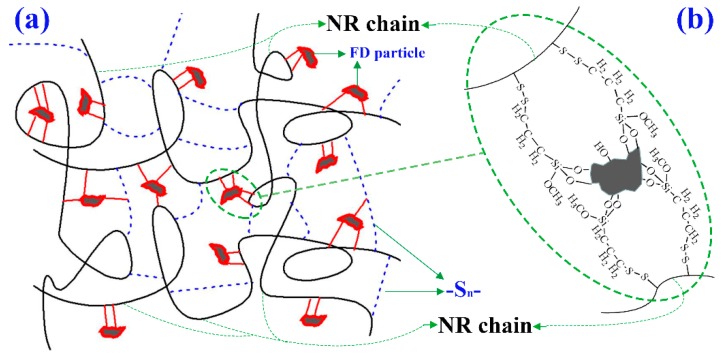
Schematic diagram of the interfacial interaction between mFD and natural rubber. (**a**) FD particles modified with Si 69 uniformly dispersed in the cross-linked natural rubber phase. (**b**) chemical bonds formed between FD surface and NR chains. Natural rubber macromolecular chains are represented by the long black irregular curves; natural rubber macromolecular chains are cross-linked by -S_n_- [-S_n_-: single sulfur bond (n = 1); double sulfur bond (n = 2) and multi sulfur bond (n > 2), is represented by the short blue dotted line]; FD particles is represented by the gray irregular polygon; the surface modified Si 69 on FD particles is represented by the red closed curve; the chemical bonds between the surface of FD particles and NR chains is represented by the short red line.

A schematic diagram of the interfacial interaction between mFD and NR molecular chain is illustrated in Figure 8. The modification of Si 69 shields the surface polar group of the FD particles, generating a non-polar organic layer on the particle surface (see Figure 3b,c). As a result, the compatibility between FD filler and NR matrix is significantly improved. Consequently, the mFD particles can be distributed homogeneously in the NR phase (see Figure 8a). The tetrasulfide bonds of Si 69 on the particle surface are broken during high temperature vulcanization and new chemical bonds (e.g., disulfide bond) form between the surface of FD filler and the mocromolecular chains of NR. [11,17].

**Figure 9:**
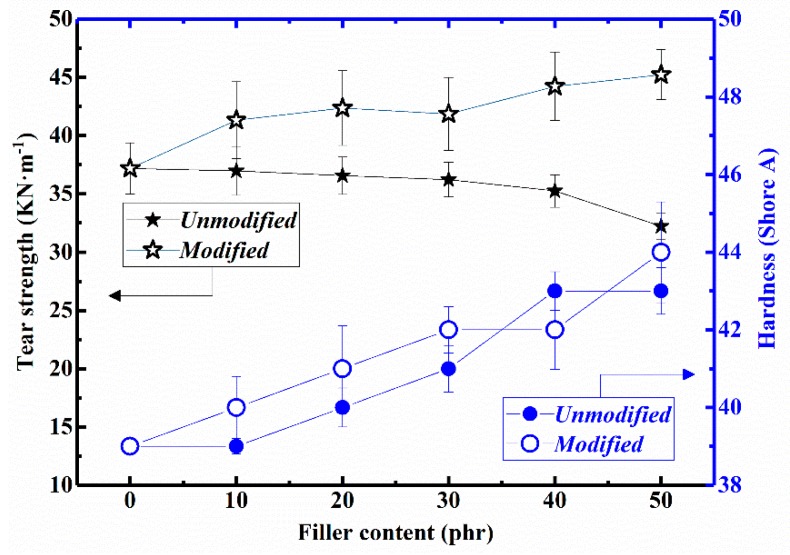
The influence of filler content on tear strength and hardness of the FD/NR and mFD/NR composites.

Tear strength and hardness of the NR composites were also measured and the results are depicted in Figure 9. The obtained tear strength and shore A hardness of pure NR are 37.19 and 39, respectively. It was observed that the tear strength of the FD/NR composite decreased slowly with increasing FD content. The tear strength of the 50 FD/NR composite is 32.23 KN·m^−1^. Compared with pure NR, it is only reduced by 13%. After modified with Si 69, the tear strength of the mFD/NR composites increase apparently with the increase of mFD content. Compared with pure NR, it is increased by about 21.3%. Additionally, the hardness of both the FD/NR composites and mFD/NR composites enhance with increasing filler content. It can be interpreted by the fact that the filler is a hard material (the main component is SiO_2_). The more fillers in the rubber, the greater the hardness of the rubber. The use of coupling agent has little effect on the hardness of rubber because it is a very thin layer of organic substance. The difference of hardness between FD/NR and mFD/NR composite with same filler content can be attributed to measurement error.

**Figure 10:**
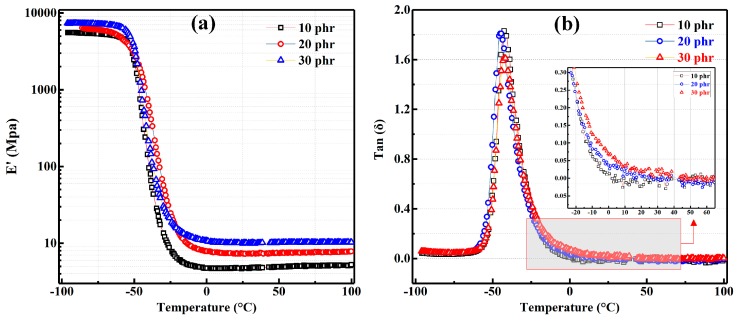
DMA measurement of the mFD/NR composites with different content of mFD. (**a**) storage modulus (E’) and (**b**) loss factor (tan δ) of the composites as a function of temperature.

Finally, we use DMA to analyze the dynamic mechanical properties of the rubber composites, and the results are shown in Figure 10. As can be seen from Figure 10a that the storage modulus (E’) of the mFD/NR composites increase with the increase of mFD content in both the low temperature range (below −50 °C) and the high temperature range (above 17 °C). As mentioned earlier, mFD is composed of a variety of oxides, in which silica content is the highest. The modulus of these oxides is much larger than that of pure NR. Therefore, the higher the content of mFD, the greater the modulus of mFD/NR composite. Important parameters, such as glass transition temperature (Tg), values of tan δ at 0 °C and 60 °C, were calculated from Figure 10b and are summarized in Table 4. The Tg of 10 mFD/NR, 30 mFD/NR and 50 mFD/NR composite are −44.5, −42.6 and −41.7 °C, respectively. As mentioned earlier (also see Figure 8b), chemical bonds between filler surfaces and the molecular chains of NR are formed during vulcanization, which immobilizes the movement of the rubber chain [14,15,27]. The movement of rubber molecular chains is increasingly restricted with increasing filler content. As a result, Tg, of the NR composite decreases with increasing filler content.

It should be mentioned that the values of tan δ at 0 °C and 60 °C are important parameters for rubber products, relating to rolling resistance and wet grip property. It is highly desirable for tire product that the rubber product simultaneously possesses low rolling-resistance and high wet grip properties [14,15,27,28,29]. In the present case, tan δ at 0 °C of the NR composite increases with increasing mFD content. The value of tan δ (0 °C) of 10 mFD/NR, 30 mFD/NR and 50 mFD/NR composite is 0.112, 0.138 and 0.169, respectively, which indicates that the wet grip property of 50 mFD/NR composite is increased by 50.9%, compared to that of 10 mFD/NR composite. In addition, the rolling-resistance of 50 mFD/NR composite shows an increment of 11.9% in comparison with that of the 10 mFD/NR composite. In conclusion, the wet grip property of 50 mFD/NR composite has been greatly improved, while the rolling resistance of the composite has a small increase, so the overall performance has been improved.

## 6. Conclusions

We have demonstrated the possibility of utilizing foundry waste derived filler for the preparation of NR based composites. The filler of FD for natural rubber were obtained through a simple, high effective, and low-cost processing method. After treated with silane coupling agent of Si 69, the mFD particles could be dispersed in NR phase homogeneously with improved microstructure. The FD and mFD powder could be easily mixed with NR in a two-roll open mill even when high filler content up to 50 phr was adapted. Tensile strength of the 50 mFD/NR composite was 15.36 MPa, which was reduced by 21% in comparison with pure NR. However, it still met the requirements for many rubber products. In addition, tear strength and hardness were improved obviously by the incorporation of mFD. Elongation at break of the composite changed a little and still remained at high value larger than 800%. Furthermore, compared to 10 mFD/NR composite, the 50 mFD/NR composite showed a large increase of wet grip property and a slight increase of rolling resistance. In light of the above investigation results, it is possible to assert that the processed foundry dust is suitable for application as filler in NR composites with acceptable mechanical properties, while lowering the cost of NR products due to the large amount of filler content. In particular, the problem of landfill of foundry waste can also be solved to a certain extent.

## Figures and Tables

**Table 1:** Compound recipe of the natural rubber (NR) composites.

Ingredient	Content (phr)	Content (phr)	Content (phr)
	Neat NR	FD/NR Composites	mFD/NR Composites
Natural	100	100	100
Zinc oxide	6	6	6
Stearic acid	3	3	3
Accelerant DM	3	3	3
Antioxidant 4010NA	1	1	1
FD	0	Varied:10, 20, 30, 40, 50	0
mFD	0	0	Varied: 10, 20, 30, 40, 50
Sulfur	4	4	4

**Table 2:** XRF analysis of the FD powder.

Analyte	Result wt.%	Analyte	Result wt.%
O	49.00		
Si	33.74	SiO_2_	72.17
Al	6.59	Al_2_O_3_	12.51
Ca	2.64	CaO	3.66
Fe	2.40	Fe_2_O_3_	3.43
Mg	1.86	MgO	3.09
Na	1.69	Na_2_O	2.28
K	1.66	K_2_O	2.00
S	0.29	SO_3_	0.64
Ti	0.13	TiO_2_	0.22
Total	100	Total	100

**Table 3:** Vulcanization parameters of the mFD/natural rubber composites.

mFD Content phr	MH dN·m	ML dN·m	(MH-ML) dN·m	tc90 s
0	0.42	0.02	0.40	286
10	0.52	0.02	0.50	281
20	0.65	0.02	0.63	272
30	0.67	0.03	0.64	263
40	0.74	0.03	0.71	251
50	0.83	0.04	0.79	245

MH: the minimum torque; ML: the maximum torque; MH-ML: the torque difference; tc10: scorch time; tc90: normal curing time.

**Table 4:** DMA measurement results of the mFD/NR composites with different filler content.

Filler Content (phr)	Tg (°C)	Tan δ at 0 °C	Tan δ at 60 °C
10	−44.5	0.112	0.092
30	−42.6	0.138	0.091
50	−41.7	0.169	0.103
